# Aqueous Humor Antioxidants in Glaucoma: Correlations With Subtypes, Intraocular Pressure, and Medication Use—A Prospective Study

**DOI:** 10.1167/tvst.14.5.7

**Published:** 2025-05-05

**Authors:** Yu-Ting Tsao, Hung-Chi Chen, Yi-Jen Hsueh, Yu-Chun Cheng, Eugene Yu-Chuan Kang, Chu-Yen Huang, Yung-Sung Lee

**Affiliations:** 1 Department of Ophthalmology, Chang Gung Memorial Hospital, Linkou Medical Center, Taoyuan, Taiwan; 2 Department of Medicine, Chang Gung University College of Medicine, Taoyuan, Taiwan; 3 Center for Tissue Engineering, Chang Gung Memorial Hospital, Linkou Medical Center, Taoyuan, Taiwan; 4 International PhD Program in Innovative Technology of Biomedical Engineering and Medical Devices, Ming Chi University of Technology, New Taipei City, Taiwan; 5 Department of Ophthalmology, Vagelos College of Physicians and Surgeons, Columbia University Irving Medical Center, New York, NY, USA; 6 Graduate Institute of Clinical Medical Sciences, College of Medicine, Chang Gung University, Taoyuan, Taiwan; 7 Department of Ophthalmology, Chang Gung Memorial Hospital, Taoyuan Branch, Taoyuan, Taiwan; 8 Department of Ophthalmology, New Taipei Municipal Tucheng Hospital, New Taipei City, Taiwan

**Keywords:** antioxidants, ascorbic acid, glaucoma, intraocular pressure

## Abstract

**Purpose:**

To investigate the correlations between aqueous humor total antioxidant capacity (TAC) and glaucoma subtypes, intraocular pressure (IOP), and glaucoma medications.

**Methods:**

This was a prospective case–control study that included 303 patients who underwent cataract surgery between April 2019 and September 2024. The participants were categorized into primary open-angle glaucoma (POAG), primary angle-closure glaucoma (PACG), neovascular glaucoma (NVG), uveitic glaucoma (UG), and control groups. Aqueous humor samples were collected at the onset of surgery, and TAC and ascorbic acid (AA) levels were measured.

**Results:**

Significant differences in TAC levels were observed among glaucoma subtypes, with UG showing the highest levels, followed by POAG, PACG, and NVG (*P* < 0.001). Multivariate linear regression further revealed that TAC levels were significantly associated with maximal IOP history (β = −0.013; 95% confidence intervals [CI], −0.023 to −0.002; *P* = 0.017) and IOP fluctuations (β = −0.016; 95% CI, −0.027 to −0.004; *P* = 0.007). However, no correlation was found between TAC levels and glaucoma medications; therefore, TAC may play a role in glaucoma pathophysiology and may serve as a potential therapeutic target.

**Conclusions:**

Glaucoma subtype and IOP dynamics significantly influenced the TAC levels of aqueous humor. Future research could target antioxidant therapies for patients with low TAC, particularly those with PACG, NVG, or a history of elevated or fluctuating IOP.

**Translational Relevance:**

This study highlights the significant variation in TAC among glaucoma subtypes and its association with fluctuating IOP and thus contributes to a deeper understanding of glaucoma pathogenesis and provides information for future therapeutic research.

## Introduction

Glaucoma, the primary cause of irreversible global blindness, exhibits an upward trend and is expected to affect over 100 million individuals by 2040.^1^ The risk factors associated with glaucoma are classified as unmodifiable and modifiable. Unmodifiable factors include advancing age, ethnicity, and a family history of glaucoma. In contrast, elevated intraocular pressure (IOP) is a major modifiable risk factor. However, not all cases of glaucoma can be effectively managed solely by controlling the IOP.^2^^,^^3^ Therefore, investigating other potential risk factors of glaucoma pathogenesis is crucial for advancing and refining therapeutic strategies.

Total antioxidant capacity (TAC) is a promising modifiable biomarker for glaucoma and offers a comprehensive evaluation of all antioxidants that mediate oxidative stress within bodily fluids.^4^^,^^5^ This assessment holds significant potential because of its intricate association with various ocular diseases such as dry eye, cataracts, corneal endothelial decompensation, macular disease, optic neuropathy, diabetic retinopathy, and glaucoma.^6^^–^^11^ Notably, the ocular antioxidant system differs significantly from that of the serum. For example, the concentration of ascorbic acid (AA), a potent antioxidant, is approximately 30 times higher in the aqueous humor than in the serum.^12^ This underscores the critical role of TAC and AA in maintaining ocular health.

Many studies have reported a correlation between TAC and glaucoma.^4^^,^^13^^–^^17^ However, few studies have focused on the correlation between TAC and various glaucoma subtypes. Within the broad spectrum of glaucoma, which includes primary and secondary subtypes, previous research has primarily focused on primary open-angle glaucoma (POAG) and pseudoexfoliation glaucoma, with little consideration to other important subtypes such as primary angle-closure glaucoma (PACG) or secondary glaucoma. Secondary glaucoma, particularly those associated with inflammatory and ischemic mechanisms, such as neovascular glaucoma (NVG) and uveitic glaucoma (UG), may demonstrate a significant association with TAC.^18^^,^^19^ Therefore, investigating the correlation between TAC and AA across various glaucoma subtypes could deepen our current understanding of their role in glaucoma.

This study aimed to investigate the correlation between glaucoma and TAC or AA to elucidate the variations in TAC and AA levels across diverse glaucoma subtypes, including POAG, PACG, NVG, and UG. A secondary objective was to examine the correlation between IOP and TAC or AA in different glaucoma subtypes. The findings of this study will enhance our understanding of the antioxidant capacity of aqueous humor in patients with glaucoma.

## Methods

### Study Population

Patients for this prospective case–control study were recruited from the Department of Ophthalmology at Chang Gung Memorial Hospital, Linkou, Taiwan, between April 1, 2019, and September 30, 2024. The study adhered to the tenets of the Declaration of Helsinki and was approved by the Institutional Review Board of Chang Gung Memorial Hospital (IRB nos. 201900017B0 and 202200547B0). Informed consent was obtained from all participants after the nature and possible consequences of the study were explained. The glaucoma group was comprised of patients diagnosed with POAG, PACG, NVG, or UG who planned to undergo cataract surgery. The control group was comprised of healthy patients scheduled to undergo cataract surgery. All participants were 18 years or older, and one eye was included per participant. Routine cataract surgery was performed by well-trained attending physicians (H-CC and Y-SL). Patients who underwent filtering surgery after standard cataract surgery were also included. Conversely, the exclusion criteria included individuals with a history of intraocular surgery, active ocular surface infections or neoplasms; those diagnosed with glaucoma subtypes other than POAG, PACG, NVG, or UG; and patients with any ocular or systemic disease that could affect optic nerve function or visual field results. The recruitment process is shown in Supplementary Figure S1, which illustrates the sequential flow of the participants throughout the study.

### Sample Collection and Data Acquisition

Aqueous humor samples were collected from each patient at the beginning of the phacoemulsification surgery, following our previously published extraction protocol.^6^^,^^7^^,^^20^ Briefly, following the creation of a side port, an irrigation tube connected to a 1-cc syringe was delicately inserted into the anterior chamber to aspirate a small volume of aqueous humor before the collapse of the anterior chamber. The undiluted samples were stored in a −80°C freezer as soon as possible until analysis.

Clinical data were extracted from electronic medical records, surgical reports, and examination findings. IOP measurements were obtained using a pneumatonometer (TX-10; Canon Corporation, Tokyo, Japan), and visual field examinations were conducted using a Humphrey Field Analyzer (Carl Zeiss Meditec, Dublin, CA). Maximal and minimal IOP measurements during clinic visits, along with IOP fluctuations (calculated as the difference between the maximal and minimal values), were documented for patients with at least three preoperative IOP readings. The visual field index (VFI) from visual field assessments conducted within 1 year prior to cataract surgery was recorded to assess the visual field status.

### TAC and AA Quantification

TAC and AA levels in the aqueous humor were quantified using established methods.^6^^,^^7^^,^^20^ For the TAC assay, 10 µL of undiluted aqueous sample was added to wells of a 96-well microplate, followed by the addition of 200 µL of 0.08% CuSO_4_ solution diluted with bicinchoninic acid (BCA). The mixture was then incubated for 20 minutes in the dark. The antioxidant compounds in the samples reduced Cu^2+^ to Cu^1+^, forming a violet chelate complex with BCA. Absorbance was measured at 570 nm using an absorbance microplate reader (Sunrise; Tecan, Männedorf, Switzerland). The detailed procedure and performance of the measurement protocol have been described in our previous study.^6^^,^^7^^,^^20^ The results of TAC were expressed as “mM AA equivalent antioxidant capacity.” Furthermore, AA concentration was measured using the OxiSelect Ascorbic Acid Assay Kit (FRASC; Cell Biolabs, San Diego, CA).^21^

### Statistical Analysis

Descriptive statistics were used to summarize the patient characteristics, with means and standard errors or proportions presented as appropriate. The Kolmogorov–Smirnov test was used to assess the normality of the distribution of continuous data. Student's *t*-test was used to compare TAC and AA levels, as well as AA:TAC ratios between the glaucoma and control groups for normally distributed data (e.g., maximum IOP in our study group). The Mann–Whitney *U* test was used for non-normally distributed data (e.g., TAC, AA levels, AA:TAC ratios, preoperative IOP, minimum IOP, IOP fluctuations, and whether the patient received a certain type of glaucoma treatment). The Kruskal–Wallis test with post hoc analysis was employed to assess variations in TAC and AA levels among different glaucoma subtypes and to examine the correlation between the number of glaucoma medications and aqueous humor TAC and AA levels, as well as AA:TAC ratios.

Univariate and multivariate linear regression analyses were conducted to assess the associations of IOP and the VFI with aqueous humor TAC and AA levels. Confounding factors such as patient age, eye laterality, sex, body mass index, hypertension, diabetes mellitus, and other underlying diseases—including dyslipidemia, heart disease, kidney disease, lung disease, liver disease, stroke, immunocompromised status, autoimmune disease, thyroid disease, and cancer—were considered and adjusted for in all multivariate regression models. Spearman's correlation analysis was used to examine the association between the VFI and TAC in the aqueous humor.

The statistical power of this study was calculated using R 4.4.1 (R Foundation for Statistical Computing, Vienna, Austria), and all other statistical analyses were conducted using SPSS Statistics 22.0 for Windows (IBM Corp., Chicago, IL). *P* < 0.05 was considered statistically significant.

## Results

### Participants

Between April 2019 and September 2024, 303 participants underwent thorough examinations and aqueous humor sample analyses. All participants underwent ocular examinations and aqueous humor aspiration without any reported adverse events. Table 1 presents the demographic and clinical characteristics of 303 participants. Significant differences were observed in the mean age (*P* = 0.001), prevalence of diabetes mellitus (*P* < 0.001), and other underlying systemic diseases (*P* < 0.001) among the groups. However, no significant differences were found regarding eye laterality, sex, body mass index, hypertension, or glaucoma severity as measured by the average retinal nerve fiber layer thickness on disc optical coherence tomography and the VFI on visual field testing. The statistical power of this study was 0.987.

**Table 1.** Baseline Characteristics of the Study Population

Characteristic	Control (*n* = 197)	POAG (*n* = 29)	PACG (*n* = 25)	NVG (*n* = 34)	UG (*n* = 18)	*P*
Eye (right/left), *n*	87/110	16/13	11/14	16/18	6/12	0.676^a^
Age (y), mean ± SE	66.51 ± 0.80	65.07 ± 2.76	68.68 ± 2.08	63.55 ± 1.70	55.67 ± 3.13	**0.001** ^b^
Sex (male/female), *n*	105/92	15/14	10/15	22/12	12/6	0.308^a^
Body mass index, mean ± SD	25.13 ± 0.26	25.48 ± 0.86	24.52 ± 0.69	24.86 ± 0.63	24.27 ± 0.70	0.915^b^
Underlying disease, *n* (%)						
Hypertension	85(43.1)	9 (31.0)	7 (28.0)	11 (32.4)	3 (16.7)	0.099^a^
Diabetes mellitus	55(27.9)	3 (10.3)	6 (24.0)	24 (70.6)	1 (5.6)	**<0.001** ^a^
Other systemic diseases^c^	85(42.6)	3 (10.3)	3 (13.0)	5 (15.2)	5 (27.8)	**<0.001** ^a^

a χ^2^ test.

b Kruskal–Wallis test.

c Other systemic diseases include dyslipidemia, heart diseases, kidney diseases, lung diseases, liver diseases, stroke, immunocompromised status, autoimmune diseases, thyroid diseases, and cancer.

*P* values less than 0.05 are presented in bold.

### Correlation Between Glaucoma and Aqueous Humor TAC

**Figure 1.**
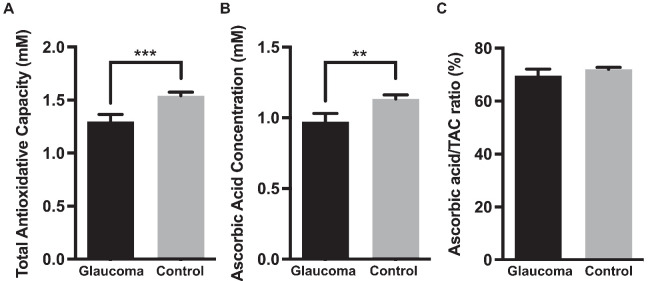
Comparative analysis of TAC, AA levels, and AA:TAC ratios between the glaucoma and control groups. (**A**) The glaucoma group exhibited a mean TAC of 1.298 ± 0.067 mM, whereas the control group demonstrated an average TAC of 1.541 ± 0.034 mM *(P* < 0.001). (**B**) The glaucoma and control groups exhibited mean AA concentrations of 0.972 ± 0.059 mM and 1.133 ± 0.029 mM, respectively (*P* < 0.001). (**C**) The glaucoma group exhibited a mean AA:TAC ratio of 69.64% ± 2.49%, whereas the control group showed 72.03% ± 0.74% (*P* = 0.077). The data are presented as mean ± SE. *P < 0.05, **P < 0.01, ***P < 0.001.

We conducted a comparative analysis of TAC and AA levels between the glaucoma and control groups. In the glaucoma group, the mean TAC was 1.298 ± 0.067 mM, with a mean AA concentration of 0.972 ± 0.059 mM and an AA:TAC ratio of 69.64% ± 2.49%. Conversely, in the control group, the mean TAC was 1.541 ± 0.034 mM, mean AA concentration was 1.133 ± 0.029 mM, and AA:TAC ratio was 72.03% ± 0.74%. Significant differences were observed in the TAC and AA levels between the two groups, whereas no significant difference was noted in the AA:TAC ratio. (*P* < 0.001 for TAC differences, *P* = 0.001 for AA differences, and *P* = 0.077 for the AA:TAC ratio) (Figs. 1A–C).

**Figure 2.**
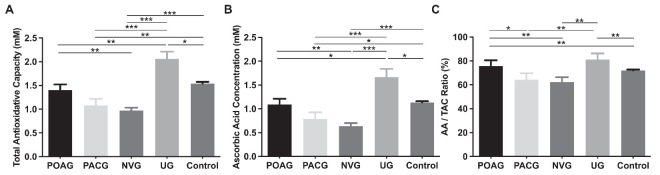
TAC, AA levels, and AA:TAC ratios among the different glaucoma subtypes. (**A**) The UG group displayed the highest average TAC levels (2.060 ± 0.154 mM), followed by the control group (1.541 ± 0.034 mM), POAG group (1.398 ± 0.122 mM), PACG group (1.079 ± 0.136 mM), and NVG group (0.970 ± 0.062 mM). (**B**) The UG group exhibited the highest mean AA concentration (1.668 ± 0.172 mM), followed by the control group (1.133 ± 0.029 mM), POAG group (1.093 ± 0.119 mM), PACG group (0.789 ± 0.137 mM), and NVG group (0.636 ± 0.065 mM). (**C**) The UG group demonstrated the highest AA:TAC ratio (81.22% ± 5.07%), followed by the POAG group (75.59% ± 4.93%), control group (72.03% ± 0.74%), PACG group (64.22% ±5.47%), and NVG group (62.41% ± 3.98%). The data are presented as mean ± SE. *P < 0.05, **P < 0.01, ***P < 0.001.

We assessed variations in TAC and AA levels among the different glaucoma subtypes and found that the UG group exhibited the highest levels, followed by the control, POAG, PACG, and NVG groups. Significant differences in both TAC and AA were observed between the POAG and NVG groups (*P* = 0.009 for TAC and *P* = 0.003 for AA), POAG and UG groups (*P* = 0.009 for TAC and *P* = 0.042 for AA), PACG and UG groups (*P* < 0.001 for both TAC and AA), PACG and control groups (*P* = 0.004 for TAC and *P* = 0.011 for AA), NVG and UG groups (*P* < 0.001 for both TAC and AA), NVG and control groups (*P* < 0.001 for both TAC and AA), and UG and control groups (*P* = 0.021 for both TAC and AA) (Figs. 2A, 2B). Additionally, regarding the AA:TAC ratio, the UG group had the highest ratio, followed by the POAG, control, PACG, and NVG groups. Significant differences in the AA:TAC ratio were noted between the POAG and PACG groups (*P* = 0.034), POAG and NVG groups (*P* = 0.001), POAG and control groups (*P* = 0.006), PACG and UG groups (*P* = 0.014), NVG and UG groups (*P* = 0.001), and UG and control groups (*P* = 0.004) (Fig. 2C).

In subsequent linear regression analyses, the different glaucoma subtypes revealed a significant association with aqueous humor TAC in both univariate and multivariate models. In the multivariate linear regression analysis, a significant difference was noted between the PACG and control groups (β = −0.60; SE = 0.13; *P* < 0.001); NVG and control groups (β = −0.47; SE = 0.11; *P* < 0.001); UG and control groups (β = 0.53; SE = 0.15; *P* < 0.001); PACG and POAG groups (β = −0.45; SE = 0.16; *P* = 0.005); NVG and POAG groups (β = −0.33; SE = 0.15; *P* = 0.033); UG and POAG groups (β = 0.67; SE = 0.18; *P* < 0.001); UG and PACG groups (β = 1.13; SE = 0.19; *P* < 0.001); and UG and NVG groups (β = 1.00; SE = 0.18; *P* < 0.001) after adjusting for all confounding factors listed in Table 1, including age, eye laterality, sex, body mass index, hypertension, diabetes mellitus, and other underlying diseases. Details of the linear regression models are summarized in Table 2.

**Table 2.** Linear Regression Analysis: Beta Coefficients and Standard Errors for TAC Across Patients With Various Glaucoma Types

	Unadjusted Model			Adjusted Model^a^		
	β^b^	SE	*P*	β	SE	*P*
Control group as baseline						
POAG	−0.14	0.10	0.169	−0.15	0.12	0.213
PACG	−0.46	0.11	**<0.001**	−0.60	0.13	**<0.001**
NVG	−0.57	0.10	**<0.001**	−0.47	0.11	**<0.001**
UG	0.52	0.13	**<0.001**	0.53	0.15	**<0.001**
POAG group as baseline						
PACG	−0.32	0.14	**0.024**	−0.45	0.16	**0.005**
NVG	−0.43	0.13	**0.001**	−0.33	0.15	**0.033**
UG	0.66	0.16	**0.003**	0.67	0.18	**<0.001**
Control	0.14	0.10	0.169	0.15	0.12	0.213
PACG group as baseline						
POAG	0.32	0.14	**0.024**	0.45	0.16	**0.005**
NVG	−0.11	0.14	0.427	0.13	0.16	0.421
UG	0.98	0.16	**<0.001**	1.13	0.19	**<0.001**
Control	0.46	0.11	**<0.001**	0.60	0.13	**<0.001**
NVG group as baseline						
POAG	0.43	0.13	**0.001**	0.33	0.15	**0.030**
PACG	0.11	0.14	0.427	−0.13	0.16	0.421
UG	1.09	0.15	**<0.001**	1.00	0.18	**<0.001**
Control	0.57	0.10	**<0.001**	0.47	0.11	**<0.001**
UG group as baseline						
POAG	−0.66	0.16	**<0.001**	−0.67	0.18	**<0.001**
PACG	−0.98	0.16	**<0.001**	−1.13	0.19	**<0.001**
NVG	−1.09	0.15	**<0.001**	−1.00	0.18	**<0.001**
Control	−0.52	0.13	**<0.001**	−0.53	0.15	**<0.001**

a Age, eye laterality, sex, body mass index, hypertension, diabetes mellitus, and other systemic diseases including dyslipidemia, heart diseases, kidney diseases, lung diseases, liver diseases, stroke, immunocompromised status, autoimmune diseases, thyroid diseases, and cancer were adjusted for in the adjusted model as the confounding factors.

b β coefficient estimate.

*P* values less than 0.05 are presented in bold.

### Correlation Between IOP and Visual Field With Aqueous Humor TAC

The correlations among maximal IOP, minimum IOP during clinic visits, IOP fluctuations, preoperative IOP, and TAC or AA levels are presented as beta coefficients and standard errors in Table 3. Multivariate analysis revealed significant negative correlations between maximal IOP during clinic visits and TAC (β = −0.013; SE = 0.005; *P* = 0.017), as well as between IOP fluctuation and TAC (β = −0.016; SE = 0.006; *P* = 0.007), after adjusting for confounding factors. These findings suggest that a higher maximal IOP and greater IOP fluctuations are associated with lower aqueous humor TAC levels. Specifically, a one-unit increase in maximal IOP was associated with a 0.013-mM decrease in TAC, whereas a one-unit increase in IOP fluctuation corresponded to a 0.016-mM decrease in TAC. However, no significant correlations were found for minimum or preoperative IOP, indicating that the negative impact on TAC may be a long-term effect rather than a reflection of recent IOP conditions.

**Table 3.** Beta Coefficients and Standard Errors From a Linear Regression Model for TAC and AA in the Aqueous Humor of Patients With Glaucoma

	Unadjusted Model			Adjusted Model^a^		
IOP (mm Hg)	β^b^	SE	*P*	β	SE	*P*
TAC in aqueous humor						
Maximal IOP during clinic visits	−0.009	0.004	**0.049**	−0.013	0.005	**0.017**
Minimal IOP during clinic visits	−0.002	0.007	0.785	0.003	0.008	0.682
IOP fluctuation	−0.011	0.005	**0.025**	−0.016	0.006	**0.007**
Preoperative IOP	0.001	0.005	0.848	−0.001	0.006	0.823
AA in aqueous humor						
Maximal IOP during clinic visits	−0.006	0.004	0.140	−0.008	0.005	0.147
Minimal IOP during clinic visits	−0.006	0.007	0.378	−0.002	0.007	0.766
IOP fluctuation	−0.007	0.005	0.182	−0.007	0.006	0.188
Preoperative IOP	0.003	0.005	0.551	0.002	0.006	0.775
AA:TAC ratio in aqueous humor						
Maximal IOP during clinic visits	−0.091	0.163	0.579	−0.018	0.216	0.935
Minimal IOP during clinic visits	−0.133	0.262	0.613	−0.092	0.321	0.775
IOP fluctuation	−0.077	0.194	0.693	−0.004	0.50	0.989
Preoperative IOP	0.170	0.180	0.347	0.100	0.236	0.674

a Age, eye laterality, sex, body mass index, hypertension, diabetes mellitus, and other systemic diseases including dyslipidemia, heart diseases, kidney diseases, lung diseases, liver diseases, stroke, immunocompromised status, autoimmune diseases, thyroid diseases, and cancer were adjusted for as the confounding factors in the adjusted model.

b β coefficient estimate.

*P* values less than 0.05 are presented in bold.

Regarding the association between visual field parameters and aqueous humor TAC, our results revealed no significant association between the VFI and aqueous humor TAC levels (Supplementary Fig. S2). Additionally, no significant correlations were noted in the univariate or multivariate linear regression analyses (*P* = 0.859 and *P* = 0.464, respectively).

### Correlation Between Glaucoma Drugs and Aqueous Humor TAC

**Figure 3.**
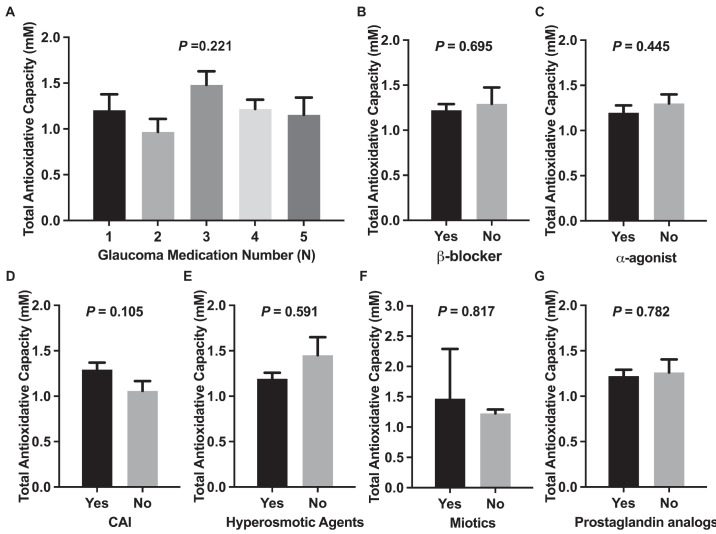
Correlation between aqueous humor TAC and number and types of glaucoma medications. (**A**) The average TAC levels are compared across patients receiving varying numbers of glaucoma medications, and no significant differences were observed among the groups. (**B**) The mean TAC level in patients who received β-blocker was 1.221 ± 0.068 mM, whereas in those who did not receive β-blocker it was 1.293 ± 0.182 mM. (**C**) The mean TAC level in patients who received α-agonist was 1.196 ± 0.082 mM, whereas in those who did not receive α-agonist it was 1.300 ± 0.099 mM. (**D**) The mean TAC level in patients who received carbonic anhydrase inhibitor was 1.293 ± 0.076 mM, whereas the level in those who did not receive it was 1.058 ± 0.109 mM. (**E**) The mean TAC level in patients who received hyperosmotic agents was 1.192 ± 0.066 mM, whereas the level in those who did not receive it was 1.452 ± 0.198 mM. (**F**) The mean TAC level in patients who received miotics was 1.471 ± 0.819 mM, whereas the level in those who did not receive it was 1.226 ± 0.064 mM. (**G**) The mean TAC level in patients who received prostaglandin analogs was 1.221 ± 0.071 mM, whereas the level in those who did not receive them was 1.262 ± 0.142 mM. The data are presented as mean ± SE. No significant correlation between aqueous humor TAC levels and various types of glaucoma medications was found.

The analysis showed no significant correlation between the number of glaucoma medications and aqueous humor TAC, AA levels, or AA:TAC ratio. Additionally, no significant differences in TAC, AA levels, or AA:TAC ratios were observed in patients receiving any type of glaucoma medication (Fig. 3, Supplementary Figs. S3, S4).

## Discussion

The present study demonstrated that TAC and AA, key reducing agents in the aqueous humor, were generally lower in patients with glaucoma, although variations existed among different subtypes. Specifically, patients with UG exhibited the highest TAC and AA levels in the aqueous humor, followed by those in the control group. Patients with POAG, PACG, and NVG displayed lower TAC and AA levels. This discrepancy suggests that the roles of TAC and AA in the pathogenesis of different glaucoma subtypes vary. Furthermore, a significant negative correlation was found between the maximal IOP during clinic visits and IOP fluctuation with aqueous humor TAC, but not with preoperative IOP. This suggests that a history of elevated IOP may lead to persistent alterations in the antioxidant levels in the aqueous humor. Additionally, no correlation was found between the type or number of glaucoma drugs used and TAC. These findings suggest that aqueous humor TAC may be influenced by glaucoma subtype, maximal IOP during clinic visits, and IOP fluctuation but not by use of glaucoma medications (Supplementary Fig. S5).

In this study, compared to the control group, patients with POAG showed no significant difference in TAC levels, which contrasts with the findings in other glaucoma groups. In addition, the patients exhibited higher AA:TAC ratios. This observation suggests that diminished TAC levels may be compensated by the increased active transport of AA into the aqueous humor, raising the possibility that TAC levels vary at different disease stages. For example, TAC may be higher in the early stages or in certain subtypes and then decline rapidly in the advanced stages. This explanation may clarify why our findings are consistent with some prior research but contradict others.^15^^,^^22^^,^^23^ Most studies have reported lower TAC levels in the aqueous humor of patients with POAG compared to those with simple cataracts (ranging from a 9% to 58% decrease, regardless of the analytical methods used). However, Ergan et al.^24^ observed an elevated total antioxidant status in patients with POAG and pseudoexfoliative glaucoma. Supplementary Table S1 lists a comparison of our findings with those of previous research on various glaucoma subtypes.^4^^,^^13^^,^^22^^,^^24^^,^^25^ Compared to other studies, our study stands out for its robust design, which includes adjustments for confounding factors and a relatively large sample size, thus enhancing the validity of our results compared to previous studies on aqueous tumor samples. However, further studies are required to confirm these findings.

Furthermore, our investigation revealed markedly decreased TAC and AA levels in patients in the PACG and NVG groups. This could be attributed to the risk of a history of high maximal IOP and IOP fluctuations, as noted in our results. Moreover, previous studies have indicated that iris damage can lead to chronic loss of antioxidant capacity.^26^ The acute onset of high IOP may exacerbate iris damage, potentially explaining the lower TAC and AA levels observed in patients with PACG and a history of high documented IOP. Similarly, patients with NVG, who present damage to the iris microenvironment and develop rubeosis, may partially account for the observed variations in TAC and AA levels. These observations raise the possibility that antioxidant therapies could be beneficial when signs of iris damage are detected or in glaucoma subtypes, such as PACG and NVG, where iris damage is likely. Future research should investigate the potential correlation between iris damage and aqueous humor TAC, as well as the efficacy of targeted antioxidant therapies in mitigating oxidative stress and preserving glaucoma progression. In contrast, although both NVG and UG were characterized by blood–aqueous barrier breakdown, UG showed the highest TAC levels and NVG exhibited the lowest. This suggests that the breakdown of the blood–aqueous barrier may not be the primary factor affecting TAC levels in the aqueous humor.

This study provides novel insights into the potential correlations among a history of high IOP, IOP fluctuation, and decrease in TAC levels in the aqueous humor. These findings offer a potential explanation for why some patients, even with well-controlled IOP over long periods, may experience ongoing deterioration in glaucoma.^27^^–^^29^ Furthermore, although most current studies focus on the impact of TAC on glaucoma progression, the efficacy of combining antioxidant therapy with glaucoma treatment remains contentious.^30^ Our study suggests that this controversy might partially stem from enrolling different target patient populations. Therefore, we hypothesized that antioxidant therapy is more beneficial for individuals who experience episodes of high IOP and difficulty regulating oxidative stress on their own. Furthermore, antioxidant therapy may be especially suitable for glaucoma subtypes, such as PACG and NVG, which are characterized by lower TAC levels. However, further clinical trials are necessary to validate these hypotheses. Additionally, our study revealed no significant correlation between the type or number of glaucoma medications and aqueous humor TAC levels. This is reasonable, as current mainstream anti-glaucoma medications primarily target aqueous humor dynamics rather than antioxidant pathways. Our study further confirmed that these medications did not significantly alter the antioxidant environment in aqueous humor. However, some anti-glaucoma therapies targeting antioxidant capacity, such as LA@VNE (a combination of latanoprost and α-tocopherol), are currently being developed.^31^ It would be valuable to investigate whether these antioxidant-targeted therapies can effectively enhance TAC levels in the aqueous humor.

The strength of this study is that we conducted TAC analysis in four important and prevalent glaucoma subtypes, three of which are infrequently reported. Moreover, we calculated statistical power and implemented rigorous methodologies, including triplicate sample analysis, to minimize bias and adjust for potential confounding factors in the regression models to mitigate selection bias. This study has certain limitations. First, a longitudinal follow-up to monitor changes in antioxidant capacity and their association with glaucoma progression was unfeasible because of the invasive nature of obtaining aqueous humor samples. Therefore, although our findings provide valuable insights, they do not establish a definitive temporal relationship between antioxidant capacity and glaucoma. To address this challenge in future research, one strategy could be to investigate the correlation between aqueous humor and serum TAC levels, allowing for longitudinal monitoring of serum TAC. In addition, the development of non-invasive optical methods to measure aqueous humor TAC could provide a promising alternative. Second, due to the limited number of cases, we did not perform matching in our study despite baseline variations within our patient group; for example, patients with UG tended to be younger, whereas patients with NVG often had diabetes mellitus. Nevertheless, we accounted for potential confounders such as age, eye laterality, sex, body mass index, hypertension, diabetes mellitus, and other underlying comorbidities by adjusting for these factors in our multivariate linear regression model. Finally, although we observed lower TAC and AA levels in patients with glaucoma than in healthy controls, the pathogenic relationship between TAC and glaucoma remains unclear, and the effectiveness of antioxidant therapies in treating or preventing glaucoma progression requires further investigation. Exploring the effect of systemic antioxidants on TAC levels in patients with glaucoma may be a valuable next step. Robust clinical trials are essential to validate the potential role of antioxidant therapies in glaucoma treatment.

In conclusion, this prospective case–control study provides valuable insights into the aqueous humor TAC and AA in different patients with glaucoma. Our findings suggest that the levels of aqueous humor TAC may be influenced by different glaucoma subtypes, peak IOP history, and IOP fluctuations but not by the type or number of glaucoma medications. This underscores the potential for the development of targeted antioxidant therapies, with future research focusing on specific glaucoma populations, such as those with PACG or NVG or individuals who have experienced high IOP episodes or significant IOP fluctuations. These findings pave the way for personalized treatment approaches that could potentially mitigate oxidative stress–related damage in patients with glaucoma, ultimately improving their clinical outcomes.

## Supplementary Material

Supplement 1

Supplement 2

Supplement 3

Supplement 4

Supplement 5

Supplement 6
