# Supplementary material for: Aqueous Humor Antioxidants in Glaucoma: Correlations With Subtypes, Intraocular Pressure, and Medication Use—A Prospective Study
Source: Transl Vis Sci Technol. 2025 May 5;14(5):7. doi: 10.1167/tvst.14.5.7 (PMC12060068; doi:10.1167/tvst.14.5.7)
Supplement: Supplement 1 [file tvst-14-5-7_s001.pdf]

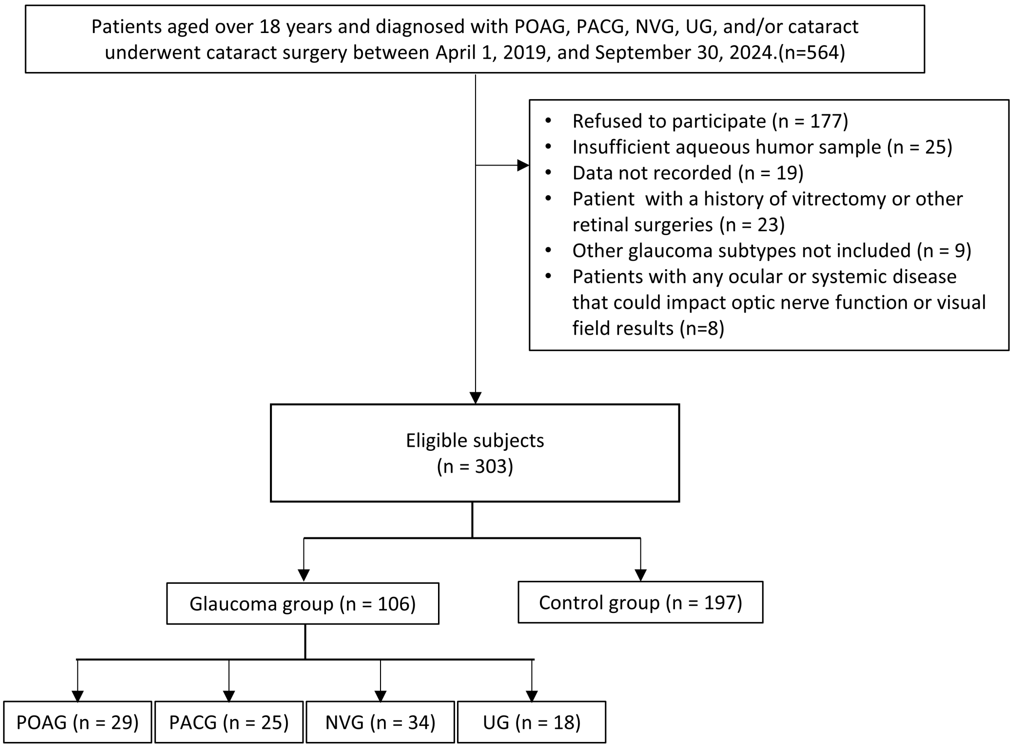

Supplementary Figure 1. Flow diagram illustrating the recruitment process of study participants.

Abbreviation: NVG = neovascular glaucoma; POAG = primary open-angle glaucoma; PACG = primary angle-closure glaucoma; UG = uveitic glaucoma
