# Supplementary material for: Aqueous Humor Antioxidants in Glaucoma: Correlations With Subtypes, Intraocular Pressure, and Medication Use—A Prospective Study
Source: Transl Vis Sci Technol. 2025 May 5;14(5):7. doi: 10.1167/tvst.14.5.7 (PMC12060068; doi:10.1167/tvst.14.5.7)
Supplement: Supplement 2 [file tvst-14-5-7_s002.pdf]

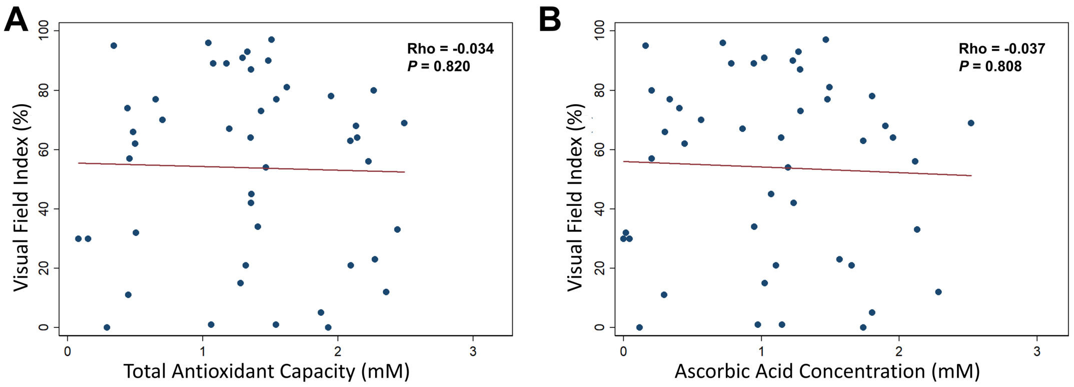

Supplementary Figure 2. The scatter plot illustrates the correlation between total antioxidant capacity (TAC) and ascorbic acid (AA) with the visual field index (VFI, %) of visual field.

(A) Spearman's correlation analysis revealed no significant correlation between TAC and VFI (Rho = -0.034, P = 0.820). (B) Spearman's correlation analysis showed no significant correlation between AA and VFI (Rho = -0.037, P = 0.808).
