# Supplementary material for: Aqueous Humor Antioxidants in Glaucoma: Correlations With Subtypes, Intraocular Pressure, and Medication Use—A Prospective Study
Source: Transl Vis Sci Technol. 2025 May 5;14(5):7. doi: 10.1167/tvst.14.5.7 (PMC12060068; doi:10.1167/tvst.14.5.7)
Supplement: Supplement 3 [file tvst-14-5-7_s003.pdf]

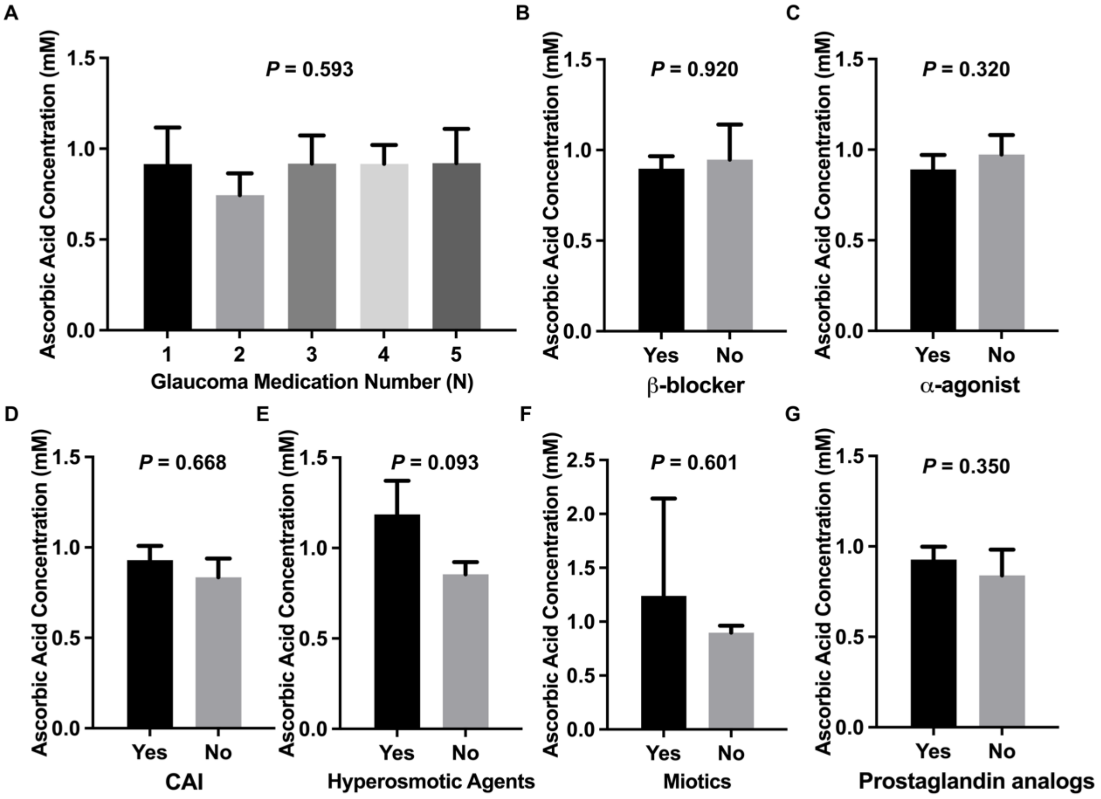

Supplementary Figure 3. The correlation between aqueous humor ascorbic acid (AA) and the number and types of glaucoma medications.

(A) The mean AA levels are compared across patients receiving varying numbers of glaucoma medications, with no significant differences observed among groups. (B) The mean AA levels in patients who received β-blocker are 0.898 ± 0.068, whereas in those who did not receive it is 0.947 ± 0.7194. (C) The mean AA levels in patients who received α-agonist is 0.891 ± 0.080, whereas in those who did not receive it is 0.974 ± 0.107. (D) The mean AA levels in patients who received carbonic anhydrase inhibitor are 0.930 ± 0.079, whereas in those who did not receive it is 0.835 ± 0.103. (E) The mean

AA levels in patients who received hyperosmotic agents are 1.186 ± 0.186, whereas in those who did not receive it is 0.855 ± 0.067. (F) The mean AA levels in patients who received miotics are 1.239 ± 0.904, whereas in those who did not receive it is 0.898 ± 0.064. (G) The mean AA levels in patients who received prostaglandin analogs are 0.927 ± 0.072, whereas in those who did not receive it is 0.840 ± 0.142. The data were presented as mean ± standard error. There is no significant correlation between aqueous humor AA levels and any of the types of glaucoma medications.
