# Supplementary material for: Aqueous Humor Antioxidants in Glaucoma: Correlations With Subtypes, Intraocular Pressure, and Medication Use—A Prospective Study
Source: Transl Vis Sci Technol. 2025 May 5;14(5):7. doi: 10.1167/tvst.14.5.7 (PMC12060068; doi:10.1167/tvst.14.5.7)
Supplement: Supplement 4 [file tvst-14-5-7_s004.pdf]

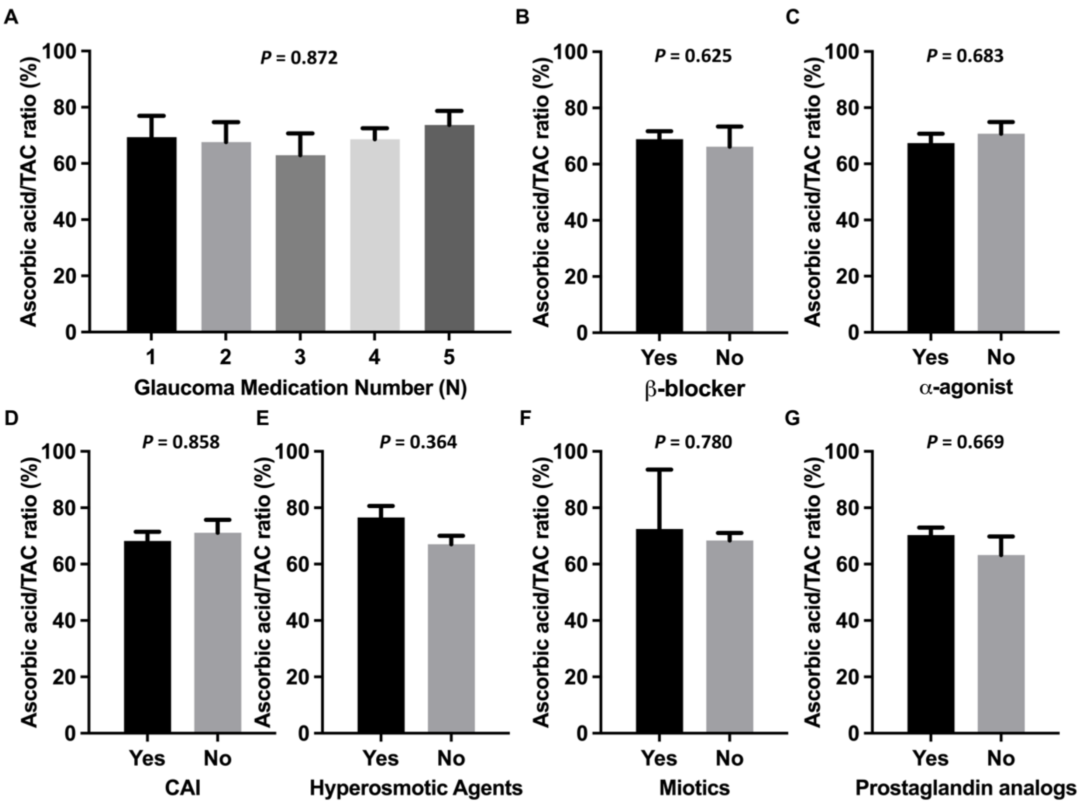

Supplementary Figure 4. The correlation between aqueous humor ascorbic acid (AA) to total antioxidant capacity (TAC) ratio and number and types of glaucoma medications.

(A) The mean AA to TAC ratio is compared across patients receiving various glaucoma medications, with no significant differences observed among groups. (B) The mean AA to TAC ratio in patients who received β-blocker is 68.90 ± 2.82%, whereas in those who did not receive it is 66.23 ± 7.14%. (C) The mean AA to TAC ratio in patients who received α-agonist is 67.42 ± 3.33%, whereas in those who did not receive it is 70.74 ± 4.17%. (D) The mean AA to TAC ratio in patients who received carbonic anhydrase inhibitor is

68.14 ± 3.22%, whereas in those who did not receive it is 71.16 ± 4.57%. (E) The mean AA to TAC ratio in patients who received hyperosmotic agents is 76.59 ± 4.08%, whereas in those who did not receive it is 67.09 ± 2.97%. (F) The mean AA to TAC ratio in patients who received miotics is 72.47 ± 21.11%, whereas in those who did not receive it is 68.44 ± 2.65%. (G) The mean AA to TAC ratio in patients who received prostaglandin analogs is 70.32 ± 2.70%, whereas in those who did not receive it is 63.21 ± 6.58%. The data were presented as mean ± standard error. There is no significant correlation between aqueous humor AA to TAC ratio and any of the types of glaucoma medications.
