# Supplementary material for: Aqueous Humor Antioxidants in Glaucoma: Correlations With Subtypes, Intraocular Pressure, and Medication Use—A Prospective Study
Source: Transl Vis Sci Technol. 2025 May 5;14(5):7. doi: 10.1167/tvst.14.5.7 (PMC12060068; doi:10.1167/tvst.14.5.7)
Supplement: Supplement 5 [file tvst-14-5-7_s005.pdf]

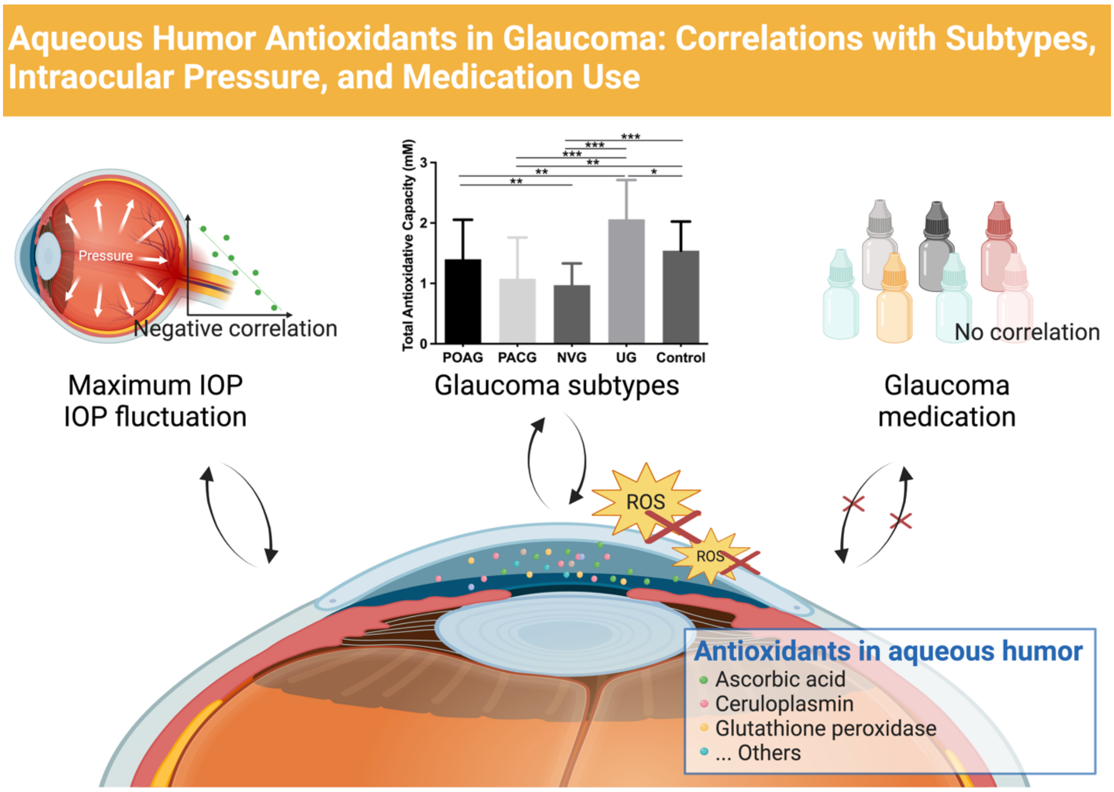

Supplementary Figure 5: Aqueous humor antioxidants in glaucoma: correlations with subtypes, intraocular pressure, and medication use.

This figure summarizes the key findings of the study, showing that the total antioxidant capacity (TAC) in aqueous humor varies across different glaucoma subtypes and is negatively correlated with both maximum intraocular pressure (IOP) and IOP fluctuations. No significant correlation was found between anti-glaucoma medications and aqueous humor TAC levels.
