# Supplementary material for: Aqueous Humor Antioxidants in Glaucoma: Correlations With Subtypes, Intraocular Pressure, and Medication Use—A Prospective Study
Source: Transl Vis Sci Technol. 2025 May 5;14(5):7. doi: 10.1167/tvst.14.5.7 (PMC12060068; doi:10.1167/tvst.14.5.7)
Supplement: Supplement 6 [file tvst-14-5-7_s006.docx]

|  |  |  | |  | | |  |  | |  | |
| --- | --- | --- | --- | --- | --- | --- | --- | --- | --- | --- | --- |
| **Supplementary Table 1.** Review of aqueous humor total antioxidant capacity reports in different glaucoma subtypes | | | | | | | | | | | |
| Reference | Glaucoma subtype | | TAC level | | Sample number | Compare to control | | | Analytical methods | |  |
| Ferreira et al., 2004 | POAG | | 52 ± 7 mol/L Trolox | | 24 | Decrease 58% | | | Colorimetric reaction (elimination of ABAP) | |  |
| Sorkhabi et al., 2011 | POAG | | 0.23 ± 0.13 mmol/lit | | 28 | Decrease 32% | | | spectrophotometric method of ABTS | |  |
| Nucci et al., 2013 | POAG | | 0.79 ± 0.35 μmol Trolox Equi/g | | 40 | Decrease 18% | | | Fluorescence method (inhibit the peroxidation of β-phycoerythrin) | |  |
| Dursun et al., 2015 | Pseudoexfoliative Glaucoma | | 0.80±0.70 mmol/L Trolox | | 26 | Decrease 48% | | | Colorimetric reaction (elimination of ABAP) | |  |
| Ergan et al., 2016 | POAG Pseudoexfoliative Glaucoma | | 2.95 ± 2.0 mmol/L Trolox  2.47 ± 0.96 mmol/L Trolox | | 15 16 | Increase 64% Increase 37% | | | Spectrophotometry | |  |
| Current Study, 2025 | POAG PACG NVG UG | | 1.398 ± 0.655 mM AAEAC  1.079 ± 0.680 mM AAEAC  0.636 ± 0.376 mM AAEAC  2.060 ± 0.652 mM AAEAC | | 29  25  34  18 | Decrease 9%  Decrease 30%  Decrease 37%  Increase 34% | | | Colorimetric reaction based on Cu^2+^ | |  |
| AAEAC: ascorbic acid equivalent antioxidant capacity; ABAP = 2,2'-azobis(2-amidopropane); ABTS = 2,2′-azinobis(3-ethylbenzothiazoline-6-sulfonic acid); NVG = neovascular glaucoma; POAG = primary open-angle glaucoma; PACG = primary angle-closure glaucoma; UG = uveitic glaucoma | | | | | | | | | | | |
